# The Brain in the Age of Smartphones and the Internet: The Possible Protective Role of Sport

**DOI:** 10.3390/brainsci15070733

**Published:** 2025-07-09

**Authors:** Laura Coco, Jonida Balla, Leonardo Noto, Valentina Perciavalle, Andrea Buscemi, Donatella Di Corrado, Marinella Coco

**Affiliations:** 1 Department of Educational Sciences, University of Catania, 95124 Catania, Italy; laura0coco97@gmail.com (L.C.); leonardo_noto@hotmail.it (L.N.); valentinaperciavalle@unict.it (V.P.); 2 Department of Education and Health, Faculty of Movement Sciences, Sport University of Tirana, 1001 Tirana, Albania; jballa@ust.edu.al; 3 Italian Holistic Body Academy, 95124 Catania, Italy; andreabuscemi@virgilio.it; 4 Department of Sport Sciences, Cittadella Universitaria, 94100 Enna, Italy; donatella.dicorrado@unikore.it

**Keywords:** smartphones, communication, physical activity, smartphone and internet addiction, self-esteem and self-control, emotional resilience, physical activity and addiction

## Abstract

Background: The widespread use of smartphones and the internet has transformed communication, but excessive use has raised concerns about smartphone and internet addiction, which can lead to psychological, physical, and social issues. The objective of this literature review is to explore the relationship between smartphone and internet addiction and physical activity, particularly focusing on whether physical exercise, especially sports, can serve as a protective factor against addiction. The review aims to examine how physical activity can reduce the negative impacts of addiction and improve overall mental health. Methods: This review synthesizes empirical research on smartphone and internet addiction and its connection to physical activity. It examines studies exploring how addiction leads to physical inactivity and how participation in physical activities, especially sports, can counteract this effect. The review also evaluates research on psychological mechanisms, such as self-esteem, self-control, and emotional resilience, that mediate the relationship between physical activity and addiction. Additionally, it discusses how sociodemographic and contextual factors influence this relationship. Conclusions: The findings consistently show an inverse relationship between smartphone and internet use and physical activity, with physical activity acting as a protective factor against addiction. Sports and other physical activities have been linked to reduced addictive behaviors, enhanced psychological well-being, and improved emotional resilience. Promoting physical activity, particularly sports, along with psychological interventions, appears to be an effective strategy for preventing and treating smartphone and internet addiction. Future research should focus on developing tailored interventions and studying diverse populations to optimize addiction prevention.

## 1. Introduction

The telephone and the internet are among the most groundbreaking technologies in the history of humankind. As soon as they were invented, these technologies revolutionized how human beings communicate, interact, and live [1]. The internet, for instance, has transformed how the world communicates and shares information, while the smartphone, with its convergence of internet usage, calling, social media, and others in one machine, has universalized these technologies in our lives [1].

Perhaps its most significant influence is the proliferation of smartphones and Internet technologies across the globe, which have become an omnipresent device across all age groups [2]. While all these technologies possess numerous positive attributes—facilitating communication, learning, entertainment, and efficiency—overuse and unregulated consumption have been found to be a disadvantage, particularly as regards their impact on health, mental capacity, and conduct, especially among adolescents and young adults [3,4,5].

Recent global statistics point towards a consistent increase in screen use. The Digital 2024 Global Overview Report states that the typical user spends around 6 h and 40 min online each day, with mobile use accounting for a considerable percentage [2]. Typical screen time in nations such as the Philippines and Brazil is over 9 h each day [1]. This rising digital saturation, particularly with the population between the ages of 16 and 24, has resulted in the diffusion of Internet and smartphone addiction—a behavioral disorder characterized by compulsive use, loss of control, and impairment of functioning [6,7].

As these devices become a part of contemporary society, there have been increasing concerns about their addictive potential [2].

Smartphone and internet addiction—characterized by compulsive use affecting daily life—have become major issues over the past few years. Smartphone and internet addiction can lead to a variety of negative psychological, physical, and social consequences, including anxiety, depression, and physical inactivity [4]. It has been shown that higher levels of smartphone and internet usage are commonly associated with lower engagement in physical activities, particularly sports, which can further aggravate the effect of addiction.

Maintenance of such behavior is due to several reinforcement mechanisms. Endless flows of notifications, algorithmically selected content, and social feedback (i.e., likes, messages) stimulate the brain’s dopaminergic reward system, that is, the mesolimbic pathway, that supports habitual use through reward-seeking processes [5,8]. This neurobiological response is very much akin to that seen in substance addiction and replicates symptoms such as craving, loss of control, and withdrawal, although formal clinical status for Internet addiction has not been awarded in most diagnostic handbooks.

Concurrently, new evidence suggests that overuse of digital technology is inversely linked to physical exercise, especially among children, teenagers, and university students [4,9]. Rising sedentary behavior, prolonged screen exposure, and substitution of physical leisure with digital entertainment have yielded considerable declines in the quantity of time spent on physical exercise and sport, leading to compromised physical well-being. This trend is concerning in light of the fact that habitual physical exercise is a firmly established protective factor against depression, anxiety, cardiovascular disease, and metabolic disorders [10].

Interestingly, both of these activities—overuse of digital media and exercise—share similar neurobiological mechanisms. While overuse of digital technology leads to abnormal stimulation of dopaminergic pathways, regular exercise activates the very same pathways in a normal, self-limiting manner, releasing dopamine and modulating emotion [11].

Studies consistently show that higher levels of screen time are related to lower levels of sports and exercise, and this can also go further to exacerbate the effects of overuse of technology [5,8].

Against such worries, the present review discusses how digital technologies, most notably smartphones and the Internet, are reshaping behavioral patterns, with special reference to their ability to undermine physical activity. It summarizes recent empirical data on smartphone and Internet addiction and tests whether participation in physical exercise, particularly sport, might be a protective factor for the adverse outcomes of hyper-consumption of digital media [10,11].

We aim to investigate whether physical exercise, particularly sport, can act as a protective factor against the negative effects of smartphone and internet addiction.

## 2. Materials and Methods

This literature review examines the relationship between smartphone and internet addiction and physical activity, specifically focusing on the role of sports participation in mitigating addictive behaviors. The methodology used to gather and synthesize relevant studies includes the following steps: A comprehensive search of peer-reviewed articles and studies was conducted across multiple electronic databases, including PubMed, Google Scholar, Scopus, and Web of Science. The search was limited to studies published in English between 2010 and 2024. The following keywords were used in various combinations: smartphone addiction, internet addiction, physical activity, sports participation, mental health, psychological resilience, self-esteem, coping strategies, and addiction prevention. Only studies related to human populations, with a primary focus on university students, adolescents, and sports professionals, were considered for inclusion.

## 3. Results

### 3.1. The Relationship Between Smartphone Addiction and Practicing Sport

The rise in smartphone usage has led to increased worry about potential addiction. Scholars debate whether or not “smartphone addiction” is a clinical addiction. Others advocate for listing it in diagnostic manuals such as the DSM, citing its compulsive nature and susceptibility to causing harm [11,12,13,14,15]. Others argue that smartphone addiction ought to be considered a manifestation of overall Internet or gaming addiction [16,17]. Generally, smartphone addiction is an uncontrollable and compulsive use of mobile devices, causing physical, psychological, or social harm [18]. Running in tandem with it is “nomophobia”, or anxiety about the absence of a mobile phone [19]. Even though there is no agreed-upon definition, its effect, particularly on the level of physical activity, has become a hot topic of study.

Different studies have set forth the negative psychological and physical consequences of smartphone addiction. Research associates overuse of smartphones with depression and anxiety [20,21,22,23], loneliness [21,24], sleep disorders [21,25,26], and low self-esteem [21,27]. These negative effects highlight the broader health effects of phone addiction.

Evidence continues to grow that phone addiction has been associated with physical inactivity. Independent studies all report again and again that higher smartphone usage is associated with less physical activity. For instance, Erdoğanoğlu and Arslan [28] found that smartphone usage in university students was inversely correlated with their physical activity. Similarly, Devran Muharremoğlu [29] found that students who participated in sports had lower levels of smartphone addiction. Kuyulu and Beltekin [30] suggested that participation in sports can decrease smartphone addiction through social support and stress relief. The authors carried out research to examine the relationship between personality traits and smartphone addiction in 239 high school students studying in a sports high school (but also other types of high schools) in Şanlıurfa, Turkey, who were asked personal information questions regarding their demographic characteristics, like whether they practiced sports and their daily use of smartphones. From the correlation analysis results, a significant difference was found between groups: smartphone addiction in students doing sports was lower than that of students who did not do sports, and the authors hypothesize that the reason may be that students practicing sports move away from the phone when they engage in sport activity, also meeting some of their needs (such as socialization, stress relief, etc.) through sports instead of smartphones.

Xiang et al. [31] found that low self-control strengthened the association between sedentary behavior and problematic smartphone use, supporting the idea that behavioral control is the most influential factor.

Meta-analyses and reviews reinforce these findings. Azam et al. [21] conducted a meta-analysis involving eight cross-sectional studies [32,33,34,35,36,37,38,39] on the relationship between physical activity, sport, and smartphone addiction in youths, observed a consistently negative association between physical activity and smartphone addiction, and stated that there is consistent support for positive outcomes of physical activity or sports participation on smartphone addiction among adolescents and youths, meaning that increasing sports participation and/or physical activity can reduce smartphone addiction; for this reason, they suggest that educational institutions use sports and exercise as an intervention strategy to deal with smartphone addiction.

Likewise, Zagalaz-Sánchez et al. [40] established that most studies indicated an inverse relationship between physical activity and smartphone use.

More general studies in various populations confirm these patterns. Pereira et al. [41] found that physically inactive students are at greater risk of smartphone addiction. Kara et al. [42] found that loneliness was positively correlated with smartphone addiction in sports science students. Tanir [43] found that female students were at greater risk of smartphone addiction but indicated that physical activity participation can lower this risk. Similarly, Buke, Egesoy, and Unver [44] reported a negative association between physical activity and smartphone addiction.

Large-scale surveys corroborate these findings. In the case of a nationally representative Korean sample, Kim and Lee [45] found that physical activity was less common among individuals at greater risk of smartphone addiction. The authors suggest, in order to prevent smartphone addiction among adolescents, participation in moderate PA for more than five days a week, vigorous PA for more than three days a week, or strength exercise for more than three days a week, since the absence of regular PA increases the probability of smartphone addiction. Finally, in this study, female adolescents comprised a higher percentage of high-risk smartphone users than male adolescents (63.2% vs. 36.8%) [45].

Wang, Li et al. [46] similarly found that during the COVID-19 pandemic, greater smartphone use was associated with less physical activity.

Some research has also explored the psychological processes that mediate the link between physical activity and smartphone addiction. Lin, Teo, and Yan [47] showed that self-efficacy mediated the link, such that more active participants are more confident in managing their phone usage. Gong et al. [48] hypothesized core self-evaluation to mediate the process, while Ke et al. [49] conducted a cross-sectional study in a sample of 650 college students from 10 colleges in Guangzhou and found partial mediation of the relationship by self-esteem. These findings reflect the complex psychological processes at play. Therefore, according to the authors, the study shows that physical activity can directly reduce smartphone addiction and also decrease smartphone addiction by indirectly improving self-esteem in college students.

Evidence drawn from sports populations provides additional evidence. Sahin Koybulan et al. [50] indicated that licensed athletes who trained regularly showed lower smartphone addiction levels than non-athletes.

Gender differences have also been found. In some studies, smartphone addiction was found to be greater in women [50,51,52]. Some studies found there were no differences by gender [28,32,35], and this suggests gender can interact with other variables such as activity type, intensity, or social setting.

Recent systematic reviews have identified the necessity of physical activity interventions. Pirwani and Szabo [53] have argued that physical activity would prevent smartphone addiction among university students, but that the relationship is relatively weaker in the absence of focused interventions.

Zhang et al. [54] found that sports interventions are effective in reducing smartphone addiction, categorizing forms of exercise into mind–body exercise and general aerobic exercise, the latter of which was more effective in reducing problematic mobile phone use.

Liu et al. [55] have demonstrated that an exercise-psychological intervention combination can effectively treat smartphone addiction.

In conclusion, most studies report a negative relationship between physical activity and smartphone addiction. Participation in sports and other physical activities appears to be a promising method for preventing or reducing smartphone addiction. However, additional longitudinal and intervention studies are required to determine causal relationships and better understand the underlying mechanisms.

### 3.2. The Relationship Between Physical Activity and Internet Addiction

The term “Internet addiction disorder” is defined by Goldeberg [56] as “a maladaptive pattern of Internet use, leading to clinically significant impairment or distress” (1996), while Saliceti [57] states that IA “recognizes the dependence of the network as a pathology, an obsessive/compulsive disorder, which drives a person to overuse of this technology and includes a wide variety of behaviours and problems with impulse control”.

Engaging in physical activities and consistent exercise is crucial for a vibrant, dynamic, and health-enhancing lifestyle [58]. The World Health Organization states that actions designed to promote higher levels of physical activity are effective and sustainable health improvement strategies [58,59]. Research has shown beneficial impacts of physical activity and regular exercise on health across various age demographics, as evidenced by epidemiological and long-term follow-up studies [58,60,61]. These studies indicate that physical activity and individual fitness levels are linked to several health benefits. These benefits can relate to (a) physical wellness, including better body makeup, more favorable lipoprotein profiles and cholesterol figures, enhanced glucose and insulin responses, reduced inflammation, lower blood pressure, improved heart functions, and balanced autonomic nervous system activity, as well as (b) enhanced and consistent mental well-being [58,62,63]. Numerous studies have established important connections between a primarily inactive way of life characterized by sitting for 4 to 8 h each day and adverse effects on both mental and physical health, covering various health metrics such as metabolic, cardiovascular, and psychological aspects [58,64].

Scholarly attention has grown in recent years to focus on the relationship between physical activity (PA) and internet addiction (IA), particularly between college and university student populations. A common conclusion in the majority of research is the negative relationship between the two variables: higher levels of physical activity are related to lower degrees of internet addiction. Apart from establishing this direct correlation, newer studies have also begun to explore the psychological, emotional, and behavioral mechanisms behind this relationship and offer valuable findings for intervention and prevention strategies [58].

Cheng et al. [65] examined the mediating role of coping styles in the relationship between physical activity and internet addiction among Chinese “post-00” college students. Their findings indicated that PA was significantly associated with adaptive coping styles (r = 0.278, *p* < 0.01) and negatively associated with IA (r = −0.236, *p* < 0.01). Coping styles were inversely associated with IA (r = −0.560, *p* < 0.01). Regression analyses also indicated that PA was negatively predicting IA (B = −0.198, *p* < 0.01) but positively predicting coping styles (B = 0.986, *p* < 0.01), and coping styles were partially mediating the relationship (48.33% of the total effect). Based on the findings, the authors implied that enhancing physical activity and coping capacity might be effective ways of reducing internet addiction among university students.

Similarly, Zhihao et al. [66] also researched the self-esteem mediation between PA and IA. They indicated that physical activity was significantly and positively correlated with self-esteem (r = 0.26, *p* < 0.001) and also negatively correlated with IA (r = −0.23, *p* < 0.001). Self-esteem was negatively related to IA (r = −0.22, *p* < 0.001) and partially mediated the PA-IA association to the tune of approximately 78.95%. In addition, gender was found to moderate the relationship, such that male and female students might react differently to physical activity interventions against internet addiction.

Based on the mediating mechanisms, Qiu et al. [67] proposed a multiple mediation model to examine how much PA affects IA. The findings indicated that physical exercise had a positive prediction for self-efficacy, psychological resilience, and self-control, respectively, which negatively predicted internet addiction behaviors in turn. Similarly, Wang et al. [68] investigated the mediating effects of loneliness and learning burnout and found that physical exercise negatively predicted IA directly and indirectly through these variables. Their mediating sequences—“physical exercise → loneliness → internet addiction”, “physical exercise → learning burnout → internet addiction”, and “physical exercise → loneliness → learning burnout → internet addiction”—accounted for high percentages (9.38%, 15.63%, and 21.88%, respectively) of the overall effect.

Yang et al. [69] conducted an umbrella review of systematic reviews and meta-analyses with the aim of assessing current evidence on the impact of different interventions on internet and smartphone addiction. With regard to sport interventions, they examined the results of nine reviews on the effects of sports interventions on IA and SA [54,55,70,71,72,73,74,75]. Among these, meta-analyses of two reviews indicated that sports interventions can significantly reduce IA levels [70,72]. Moreover, network meta-analysis highlighted that the single sport, team sport, double sport, team + double sport, and team + double + single sport significantly improved IA, with the double sport being the best form of intervention [54]. Finally, three reviews considered sports interventions but did not analyze the effects individually, therefore not allowing any definitive conclusions to be drawn [71,73,74]. The authors say their study provides consistent evidence supporting the use of sports interventions in reducing IA and add that sports interventions may be a more effective treatment option than conventional treatments (e.g., psychotherapy and medication) because of the positive correlated aspects of physical activity in the treatment of IA (better sleep quality, increased blood and oxygen supply to the brain, improved neurological function, and consequent improved physical function and mental adaptability), citing Chen [76]. However, the authors also point out the limitation in the number of available studies and the lack of in-depth explorations of the influence of exercise intensity and duration, suggesting that future research should focus on establishing a uniform classification of exercise duration and investigating the effects of different exercise intensities on IA.

Xu and Tang [77] also examined the chain mediation model from self-control to loneliness in the association between physical activity and problematic internet use. Their evidence supported that physical activity might have a direct effect and an indirect effect on problematic internet use by establishing self-control and transcending loneliness, i.e., self-regulatory capacity and emotional well-being are important mediators.

Emotional and psychological mechanisms also appear to be crucial to explain the PA–IA relationship. Jelleli et al. [78] investigated the intercorrelations among internet addiction, depression, anxiety, stress, and physical activity in Tunisian university students. The findings established that PA was a mediator of negative emotional states and IA, underpinning the mental health benefits of exercise. Liu et al. [79] also investigated the mediating roles of anxiety and inhibitory control in adolescents. They discovered PA was negatively associated with anxiety and IA, whereas anxiety was positively associated with IA. Physical activity directly and indirectly predicted reduced IA through enhanced anxiety management and inhibitory control abilities.

Additionally, Liu et al. [80] explored the moderating role of PA on alexithymia, IA, and depression associations. Their findings indicated alexithymia to be positively related to IA and depression but negatively related to PA. Further, IA was discovered to mediate the relationship between alexithymia and depression, while physical activity inhibited the predictive effect of IA on depression. This highlights the importance of physical activity not only as a buffer for IA but also as a moderator for its psychological consequences.

Sociodemographic and contextual variables have also been addressed. Cai et al. [81] investigated the influence of academic year and regional differences on IA among Chinese university students and concluded that freshmen were particularly vulnerable to internet addiction. Moreover, students who engaged in recreational sport activities reported significantly lower IA scores, suggesting that physical activity acts as a protective factor in early university life. Toselli et al. [82], in their investigation of Italian teenagers, reported that sportive teenagers had lower levels of IA than their non-sportive counterparts, validating the protective role of PA in various cultural contexts.

Contradictory findings were reported by Zalewska et al. [83], who investigated Polish and Portuguese students in the final year of the COVID-19 pandemic. They found that students who had been infected with COVID-19, particularly more than once, showed a greater tendency towards IA, even among those who were physically active. These results suggest that pandemic-related stressors may moderate the buffering effects of physical activity on IA. Haug et al. [84] studied Norwegian university students and found a weak but significant bidirectional negative correlation between PA and problem gaming behavior in early pandemic stages, whereas other temporal correlations were not significant.

Similar studies on adolescents also support the reverse correlation between PA and IA. Shristi, Kaphle et al. [85] had reported that internet-addicted adolescents were physically less active. Gen et al. [86] repeated the finding among a sample of Turkish young people, and higher IA scores were associated with lower levels of PA among university students.

Taken together, the reviewed studies consistently support the finding that physical activity is a significant protective factor for internet addiction. PA appears to influence IA directly and indirectly through the enhancement of psychological traits such as self-esteem, coping, self-control, and resilience and indirectly through lowering negative emotional conditions such as loneliness, anxiety, stress, and depression. Moreover, sociodemographic and contextual factors, such as gender, study year, and pandemic stress, may moderate the direction and magnitude of this association. In general, these findings underscore the importance of promoting physical activity as a general intervention strategy to not only reduce internet addiction but also enhance overall aspects of mental health and well-being in young adults that are struggling with increasingly digitalized environments.

### 3.3. The Relationship Between the Brain, Smartphone Addiction, Internet Addiction, and Physical Activity

The rise of internet addiction (IA) and smartphone addiction (SPA) is a global phenomenon, especially prevalent among teenagers and young adults [87,88,89]. These addictions are causing considerable transformations in individuals’ lives and often have a substantial impact on their biopsychosocial well-being [90,91].

The brain’s structural and functional features linked to the overuse of the internet have garnered significant research interest over the last ten years.

Sadeghi et al. [92] examined the connection between internet addiction tendency (IAT) scores and the volumes of gray and white matter in specific regions (rGMVs and rWMVs), as well as brain activity during a working memory task within a significant cohort of healthy young individuals (*n* = 1154, average age 20.71 ± 1.78 years). A notable positive association was identified between the IAT score and the gray matter volume (GMV) of the right supramarginal gyrus (rSMG), alongside significant negative relationships with the white matter volume (WMV) in the right temporal lobe (including sub-gyral and superior temporal gyrus), right sublobar region (encompassing extra-nuclear and lentiform nucleus), the anterior lobe of the right cerebellum, the cerebellar tonsil, and areas within the right frontal lobe (specifically the inferior frontal gyrus and sub-gyral regions), as well as the pons [92]. Additionally, there was a significant positive correlation between IAT scores and brain activity in the default-mode network (DMN), medial frontal gyrus, medial superior frontal gyrus, and anterior cingulate cortex during a 2-back working memory task [92].

Furthermore, numerous studies indicate that increasing internet addiction among individuals leads to greater social isolation. On the other hand, it has been proposed that having strong social networks can shield individuals from internet addiction.

Matsunaga et al. [93] examined the brain structures of individuals in the typical adolescent age range of 10 to 18 years, proposing that the dimensions of the left dorsolateral prefrontal cortex, which is believed to be linked to self-control, may relate to both internet addiction and social capital [93]. Analyzing brain scans using voxel-based morphometry revealed a negative correlation between the volume of the left DLPFC and the intensity of internet addiction, as well as a positive correlation with social capital [93]. Additionally, correlation assessments indicated that internet addiction severity and social capital were inversely related [93]. The statistical link between these factors lost significance when the volume of the left DLPFC was accounted for as a control variable [93]. These findings imply that the left DLPFC might play a mediating role in linking social capital and internet addiction among adolescents [93].

Tseng et al. [94] created a task related to internet usage that involved a stop-signal approach, utilizing electroencephalography (EEG) to explore the concept of inhibitory control. The study involved both healthy individuals and those suffering from Internet addiction, who were engaged in the internet-related stop-signal task while their brain activity was monitored through 19-channel EEG recordings [94]. The analysis focused on the resulting event-related potentials and spectral changes. Findings indicated that participants with Internet addiction exhibited a heightened Stop-P3 response during tasks requiring inhibitory control, implying a difference in the neural mechanisms underlying their ability to manage impulsivity compared to the healthy control group [94].

Moreover, individuals suffering from Internet addiction displayed heightened low-frequency synchronization along with reduced alpha and beta desynchronization in the middle and right frontal areas when compared to individuals without the condition [94]. Irregularities in brain effective connectivity were also found, characterized by increased connections between occipital and parietal regions as well as intra-occipital links, while connections in the frontal-paracentral area were diminished in those with Internet addiction [94]. These findings indicate that physiological indicators play a crucial role in future cognitive assessments of Internet addiction to further explore the underlying processes and reliable biomarkers [94].

León Méndez [95] undertook a thorough review aiming to offer an extensive synthesis of the existing literature regarding the distinct negative impacts on cognitive functions in adolescents and young adults affected by Internet Addiction (IA) and Smartphone Addiction (SPA), utilizing functional magnetic resonance imaging (fMRI) for analysis [95]. Generally, studies utilizing neuroimaging have uncovered a developing pattern of widespread alterations in brain activity among individuals suffering from IA and SPA [95]. Young adults and adolescents dealing with these addictions tend to exhibit both functional and resting-state brain irregularities when compared to control groups [95]. These abnormalities include deficits in reward processing (involving regions such as the striatum, anterior accumbens cortex, insula, and amygdala) as well as in executive functions (linked to the dorsolateral prefrontal cortex and various lobes, including frontal, parietal, and occipital) [95]. This body of evidence may assist in pinpointing functional deficits related to IA and SPA while also emphasizing the shared and distinctive neuroanatomical features of these behavioral addictions [95].

While the research of Li et al. [96] reviewed the neurobiological mechanisms of exercise-based interventions against internet addiction. The results they obtained strengthen the idea that exercise-based interventions can be effective in treating internet addiction: exercise can increase the levels of neurotrophic factors, cortisol, and neurotransmitters; stimulate hippocampal neurogenesis; protect the autonomic nervous system; and control the reward urge; hence, the authors conclude that exercise mitigates internet addiction by regulating the neurobiology of the central and autonomic nervous systems, thus constituting a recommended intervention for internet addiction.

## 4. Discussion

### 4.1. Summary of Key Findings

The literature review presents strong support that smartphone and internet addiction is inversely associated with physical activity, including sport participation. Key findings indicate a positive correlation where higher smartphone and internet use correlate with lower levels of physical activity, and sporting participation can play a protective function in reducing the likelihood of smartphone and internet addiction. Psychological mechanisms such as self-esteem, self-regulation, and emotional strength were shown to mediate the exercise–addiction relationship. Exercise-based interventions, particularly sports-based interventions, were also shown to have efficacy in reducing addiction symptoms and improving mental well-being. All the studies seem to suggest that exercise lowers anxiety, depression, loneliness, and other negative emotional states often exacerbated by overuse of screens.

### 4.2. Synthesis and Critical Analysis

The investigated studies show several important tendencies but also are diverse. An adverse correlation between physical activity and addiction to mobile phones or the internet is detected by most studies, with higher levels of physical activity connected to decreasing degrees of addiction. However, the correlation diverges depending on varying study subjects, a measurement of physical activity, and techniques. Cross-sectional studies are more common than longitudinal studies, which limits our understanding of causal effects. Furthermore, while the majority of studies employ self-reporting measures of physical activity and smartphone use, this can introduce biases, for instance, social desirability bias, on outcomes.

There is heterogeneity in measuring addiction and physical activity as well. For instance, while some studies define smartphone addiction on clinical criteria (e.g., DSM), others use behavioral markers or screen time thresholds. Similarly, physical activity is sometimes quantified as total exercise or participation in organized sports, but intensity and duration of activity are rarely controlled. These variations in measurement make it difficult to compare outcomes between studies and draw firm conclusions.

Furthermore, most research has shown that there is a negative correlation between physical activity and addiction, but some research has shown no significant association. Gender, sociodemographic variables, and situational factors, such as the COVID-19 pandemic, appeared to moderate such associations. This suggests further research to explore such moderating variables and their influence on the relationship between physical activity and addiction.

### 4.3. Theoretical Implications

The findings based on the literature examined have several implications for theories and models currently existing in the field. The correlation between smartphone addiction and physical inactivity aligns with the Social Cognitive Theory, wherein self-regulation and self-efficacy are emphasized as being critical determinants of behavior change. Physical exercise is suggested to enhance self-esteem, self-control, and coping skills, all of which are critical in averting addiction-prone behavior. These findings also complement the Theory of Planned Behavior, where physical activity can be considered an influence on both intention and behavior in relation to smartphone usage.

Further, the Stress-Buffering Model of social support may be used to include physical exercise as a buffering support in combating the negative effects of smartphone addiction because physical exercise has been known to relieve stress and promote good mental health. The increased evidence supporting the involvement of physical activity in addiction management forms the basis for using physical activity interventions within existing psychological models of managing addiction.

### 4.4. Practical Implications

The use of the findings is applied specifically in developing interventions for internet and smartphone addiction. The results show that incorporating physical activity, particularly sports, in prevention and treatment interventions for addiction can be a very effective method. With smartphone and internet addiction on the rise, interventions promoting regular physical activity may help reduce symptoms of addiction, improve mental health, and promote overall well-being.

For instance, school-based prevention and college health programs could promote exercise by encouraging participation in sports or group exercises as a protective intervention. Mental health professionals working with individuals experiencing addictions can choose to incorporate exercise within the therapy to increase emotional stability, reduce stress, and treat symptoms of anxiety and depression.

Second, policymakers can make use of these findings to advertise physical activity programs and interventions incorporating youth into sport, particularly as a means to prevent addiction problems. Public campaigns emphasizing the emotional and mental benefits of exercise and attempts to limit screen time can help counteract the rising tide of smartphone and internet addiction.

In summary, the literature reviewed unequivocally indicates that physical activity, especially sports participation, is a protective factor against smartphone and internet addiction. Psychological mechanisms such as enhanced self-esteem and emotional resilience are key to understanding this association. While the findings are promising, more rigorous research is needed to determine causal relationships, develop standardized measures, and investigate the role of the moderating factor. Ultimately, promoting sport as a form of physical activity offers a real and effective solution to prevent as well as treat smartphone and internet addiction.

In conclusion, there is significant evidence for a negative relationship between physical activity and both smartphone and internet addiction. There are several studies that consistently show that higher levels of physical activity, particularly in the form of sports, are associated with lower smartphone addiction [27,28,97]. Physical activity appears to mitigate the adverse consequences of smartphone addiction through a number of psychological mechanisms, including improved self-esteem, improved self-control, and improved emotional resilience [47,48]. Physical activity has also been shown to alleviate symptoms of depression, anxiety, and loneliness that become worse with excessive screen time and physical inactivity [78,79].

Evidence regarding internet addiction (IA) also underscores the protective role of physical activity. Experiments conducted among university students and adolescents have shown that physical activity not only reduces IA but also enhances psychological variables such as coping capacity and self-efficacy, which further reduce addiction-prone behavior [66,76]. Additionally, the relationship between physical activity and addiction has been determined to be mediated by psychological resilience, self-control, and reduced loneliness, suggesting that physical exercise can promote psychological as well as emotional flourishing [67,68].

However, there are still large gaps in the literature requiring more research. Although the majority of studies document a negative association between physical activity and smartphone or internet addiction, further longitudinal and intervention-based research is needed to clarify the causal processes and long-term consequences of physical activity in decreasing these addictive behaviors. Gender, sociodemographic traits, and contextual variables, such as the pandemic-related stressors, may also potentially moderate the magnitude of these associations, illustrating the significance of context-specific interventions [81,83].

Moreover, the results obtained by Li et al. [96] strengthen the idea that exercise-based interventions can be effective in treating internet addiction: exercise can increase the levels of neurotrophic factors, cortisol, and neurotransmitters; stimulate hippocampal neurogenesis; protect the autonomic nervous system; and control the reward urge. Therefore, carrying out physical activity mitigates internet addiction by regulating the neurobiology of the central and autonomic nervous systems, thus constituting a recommended intervention for internet addiction.

### 4.5. Limitations

Although the literature reviewed offers useful information, there are some limitations that must be noted. The majority of the reviewed studies are cross-sectional, and therefore, it is challenging to conclude anything regarding causality. It would be necessary to use longitudinal studies or randomized controlled trials (RCTs) to be able to explain the causal relationship between physical activity and smartphone/internet addiction.

Second, the measurement of addiction and exercise varies across studies. Uniform definitions and measures would make the studies more comparable and allow stronger syntheses of findings. The limitation that can have an influence on the accuracy of the findings is the employment of self-reporting questionnaires that are vulnerable to biases.

Furthermore, the main focus of the study is young people. It is unclear whether the findings are applicable to other age or professional groups, for example, office workers and young workers. The majority of studies provide little data on the impact of the type of aerobic or strength sport, namely team or individual.

Moreover, the main focus of the study is young people. It is unclear whether the findings are applicable to other age or professional groups, for example, office workers and young workers. The majority of studies provide little data on the impact of the type of sport, namely team or individual, aerobic or strength. From the literature it would seem that it is aerobic sport (individual or team) that plays a protective role.

In addition, though the review hints at the addictive protection of exercise, the underlying psychological mechanisms that account for the link between the two are as yet unclear. Additional research must be conducted to explore how physical activity of a particular type (e.g., aerobic exercise, strength training, team sports) influences symptoms of addiction and to determine which psychological traits mediate or moderate those influences.

Lastly, the impact of sociodemographic factors, such as age, gender, and culture, was not properly investigated in most of the studies. Future research must investigate how these factors influence the relationship between physical activity and addiction to develop more specific and efficient interventions, providing clear standardized recommendations on the type of physical activity to practice to combat smartphone and internet addiction.

Furthermore, contextual factors, such as COVID-19 pandemic stressors, can impact the relationship between physical activity and addiction, and these factors need to be explored further.

## 5. Conclusions

In conclusion, promotion of physical exercise, particularly sports, appears to be an effective strategy in preventing smartphone and internet addiction. Physical exercise combined with psychological interventions has been shown to produce promising outcomes in preventing addiction symptoms and enhancing overall mental well-being. Future research must examine these mechanisms in more diverse populations with an emphasis on developing specific interventions that involve physical exercise for preventing smartphone and internet addiction.
